# Respiratory Microbiome of Carbapenem-Resistant *Acinetobacter baumannii* Ventilator-Associated Pneumonia: A Pilot Study from the Republic of Korea

**DOI:** 10.3390/pathogens14111141

**Published:** 2025-11-11

**Authors:** Se Ju Lee, Jaeeun Seong, Jung Ah Lee, Yongseop Lee, Jung Ho Kim, Jin Young Ahn, Nam Su Ku, Jun Yong Choi, Joon-Sup Yeom, Su Jin Jeong

**Affiliations:** 1 Division of Infectious Diseases, Department of Internal Medicine and AIDS Research Institute, Yonsei University College of Medicine, Seoul 03722, Republic of Korea; playit@inha.ac.kr (S.J.L.); sjaeeun1127@yuhs.ac (J.S.); peacefulee@yuhs.ac (J.A.L.); yslee@yuhs.ac (Y.L.); qetu1111@yuhs.ac (J.H.K.); comebacktosea@yuhs.ac (J.Y.A.); smileboy9@yuhs.ac (N.S.K.); seran@yuhs.ac (J.Y.C.); joonsup.yeom@yuhs.ac (J.-S.Y.); 2 Division of Infectious Diseases, Department of Internal Medicine, Inha University College of Medicine, Incheon 22332, Republic of Korea

**Keywords:** carbapenem-resistant *Acinetobacter baumannii*, microbial diversity, respiratory microbiome, ventilator-associated pneumonia

## Abstract

Ventilator-associated pneumonia (VAP) is one of the most common hospital-acquired infections. Several studies have explored the potential role of the lung microbiome as a biomarker for identifying and predicting the prognosis of VAP. However, research on the respiratory microbiome in individuals with VAP caused by carbapenem-resistant *Acinetobacter baumannii* (CRAB) remains limited. Therefore, we aimed to analyze the respiratory microbiome of patients with CRAB VAP. Respiratory specimens were collected from patients who developed CRAB VAP. Microbiome diversity and composition were analyzed using 16S rRNA gene pyrosequencing. Patients were categorized into two groups based on mortality outcomes: intensive care unit (ICU) mortality or 28-day mortality after ICU discharge. Twenty patients with CRAB VAP were enrolled, including nine in the mortality group. No significant differences were observed in α-diversity indices between the study groups. However, multivariable Firth’s logistic regression revealed a significant association between a relative abundance of the *Enterococcus* genus ≥ 1% and mortality outcomes (odds ratio: 0.06; 95% confidence interval: 0.00–0.771; *p* = 0.029). This study characterized the respiratory microbiome of patients with CRAB VAP and highlighted the potential role of microbiome analysis in predicting disease prognosis. Further studies with larger sample sizes are warranted to validate these findings.

## 1. Introduction

Ventilator-associated pneumonia (VAP) is one of the most common hospital-acquired infections within intensive care unit (ICU) [1,2]. Its incidence ranges from 2 to 16 episodes per 1000 ventilator days, depending on the study, with an attributable mortality rate of approximately 10% [1,3,4]. Delayed administration of antimicrobial agents for VAP is associated with a worse prognosis [1,5]. Therefore, early identification of VAP is crucial, and several biomarkers have been proposed for this purpose [1].

*Acinetobacter baumannii*, one of the “ESKAPE” organisms (*Enterococcus faecium*, *Staphylococcus aureus*, *Klebsiella pneumoniae*, *Acinetobacter baumannii*, *Pseudomonas aeruginosa*, and *Enterobacter* species), is a major pathogen responsible for nosocomial infections worldwide, often exhibiting multidrug resistance [6,7]. This bacterium often exhibits a highly drug-resistant phenotype, including resistance to carbapenems. Carbapenem-resistant *A. baumannii* (CRAB) is associated with high significant attributable mortality and increased medical costs [8,9]. CRAB is also a leading cause of VAP; notably, it was reported as the most frequent pathogen for this condition in the Republic of Korea [1,5,10]. Similar to other infectious diseases caused by this pathogen, patients with CRAB VAP tend to have a poor prognosis [11,12].

The lung microbiome plays a role in respiratory diseases, such as asthma and chronic obstructive pulmonary disease. Additionally, several studies have examined changes in the lung microbiome in pneumonia [13]. In particular, research has explored the potential role of the respiratory microbiome as a biomarker for diagnosing and predicting the prognosis of VAP [13,14]. However, studies on the respiratory microbiome in CRAB VAP remain limited. Notably, no research has yet investigated the characteristics of the respiratory microbiome in relation to the prognosis of CRAB VAP. Therefore, this study aimed to analyze the respiratory microbiome in CRAB VAP and identify microbiome characteristics associated with prognosis in this difficult-to-treat infection.

## 2. Materials and Methods

### 2.1. Study Design

This retrospective study enrolled patients who developed CRAB VAP and required mechanical ventilation in the medical intensive care unit of a 2400-bed tertiary hospital in the Republic of Korea. The inclusion criteria were based on the definition of VAP [15]: (1) mechanical ventilation for more than 48 h; (2) the presence of a new or progressive radiographic infiltrate; (3) at least two of the following three clinical features—body temperature > 38 °C or <36 °C, leukopenia or leukocytosis, or purulent secretions; and (4) detection of CRAB in an endotracheal aspirate culture (threshold: ≥10^5^ CFU/mL). The exclusion criteria included age < 18 years and the detection of any other pathogen in microbiological studies, including the same endotracheal aspirate culture. Patients with CRAB VAP were categorized into two groups based on composite mortality outcomes, defined as ICU mortality or death within 28 days after ICU discharge. All relevant clinical and laboratory data were retrieved from electronic medical records on the day of sample collection. The severity of illness was assessed using the Sequential Organ Failure Assessment (SOFA) score. This study was approved by the Institutional Review Board of Yonsei University College of Medicine (4-2024-1651) and conducted in accordance with the ethical standards outlined in the 1964 Declaration of Helsinki and its subsequent amendments. Because the study was retrospective and the data were anonymized, the IRB waived the requirement for informed consent.

### 2.2. Respiratory Specimen Collection

For respiratory microbiome analysis, stored respiratory specimens previously collected for microbiological tests were used. Respiratory specimens were collected through endotracheal aspiration from patients with CRAB VAP. The samples were then stored at –8 °C.

### 2.3. DNA Extraction and 16S rRNA Sequencing

Genomic DNA was isolated from endotracheal aspirate samples using the FastDNA^®^ Spin Kit for soil (MP Biomedicals, Santa Ana, CA, USA) following the manufacturer’s instructions for 16s rDNA pyrosequencing. DNA concentration was measured using an Epoch™ spectrophotometer (BioTek, Winooski, VT, USA). The quality of the extracted DNA was assessed using 1% agarose gel electrophoresis.

For 16S rRNA gene sequencing, primers targeting the V3–V4 regions were used to amplify the 16S rRNA gene: 341F (5′-TCGTCGGCAGCGTC-AGATGTGTATAAGAGACAG-CCTACGGGNGGCWGCAG-3′) and 805R (5′-GTCTCGTGGGCTCGG-AGATGTGTATAAGAGACAG-GACTACHVGGGTATCTAATCC-3′), where underlined sequences represent the target regions. Polymerase chain reaction (PCR) amplification was performed under the following conditions: an initial denaturation at 95 °C for 3 min, followed by 25 cycles of 95 °C for 30 s, 55 °C for 30 s, and 72 °C for 30 s, with a final extension at 72 °C for 5 min and an indefinite hold at 4 °C. Following this, a second PCR (index PCR) was performed using index primer pairs, including adapter I5 and adapter I7 primers. The second PCR followed the same conditions as that of the first but with the number of cycles reduced to eight.

The quality of the final library was assessed using a Bioanalyzer 2100 (Agilent Technologies, Palo Alto, CA, USA). Non-target short fragments were removed using CleanPCR (CleanNA, Waddinxveen, The Netherlands). The final library products that passed the quality control were sequenced at CJ Bioscience, Inc. (Seoul, Republic of Korea) using the MiSeq Reagent Kit v2 (500 cycles) on the Illumina MiSeq sequencing platform (Illumina, San Diego, CA, USA).

### 2.4. Diversity Assessment

The α-diversity of the respiratory microbiome was assessed using standard diversity indices. Species richness was assessed using the observed operational taxonomic unit (OTU) counts, as well as the Chao1 and abundance-based coverage estimator (ACE) indices. The Shannon and Simpson indices were used to assess species richness and evenness. These analyses were conducted using EzBioCloud’s (CJ Bioscience) Microbiome Taxonomic Profiling platform, based on OTUs obtained from pyrosequencing data. To evaluate β-diversity, we examined differences in taxonomic composition between the study groups. The results were visualized using principal coordinate analysis (PCoA) based on Bray–Curtis similarity matrices.

### 2.5. Statistical Analysis

The study population was classified based on the composite outcome of ICU mortality or 28-day mortality after ICU discharge. Differences in patient characteristics and outcomes between the groups were assessed using the Wilcoxon rank-sum test for continuous variables and the chi-square test or Fisher’s exact test for categorical variables. Kaplan–Meier analysis and the log-rank test were used to estimate 28-day mortality after the diagnosis of CRAB VAP. Firth’s logistic regression analysis was performed to assess the factors associated with the composite outcome. Variables with a *p*-value of <0.1 in the univariate analysis and deemed clinically relevant were included in the multivariable model. Statistical significance was set at a *p*-value of <0.05. All statistical analyses were performed using R version 4.0.5 (The R Foundation for Statistical Computing, Vienna, Austria).

## 3. Results

A total of 20 patients with CRAB VAP were enrolled between March 2018 and June 2022. The median age of the study population was 69.0 years (interquartile range [IQR]: 61.5–79.0 years), and 13 patients (65%) were men (Table 1). The median duration of mechanical ventilation at the time of respiratory specimen collection was 14.0 days (IQR: 9.0–31.5 days). The ICU mortality rate was 25.0%, while the in-hospital mortality rate was 55.0%. Nine patients (45.0%) died either during ICU care or within 28 days after ICU discharge, which was the primary outcome of this study. The mortality group had a higher Charlson comorbidity index (median 7.0 [IQR: 6.0–9.0] vs. 3.0 [IQR: 2.5–5.0]; *p* = 0.024) and a lower lymphocyte count on the day of respiratory sample collection (600.0/μL [IQR: 260.0–1010.0] vs. 1000.0/μL [IQR: 915.0–1525.0]; *p* = 0.046) than those of the survival group. No significant differences were observed between the two groups in terms of comorbidities, SOFA scores, or antibiotics administered on the day of sample collection.

**Figure 1:**
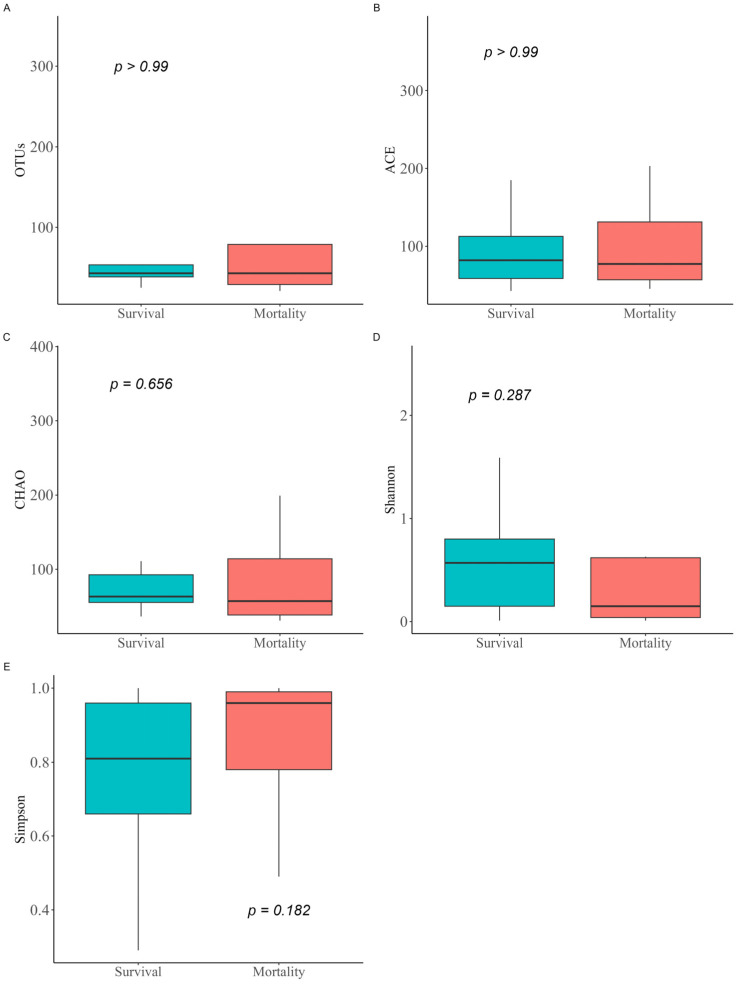
Comparison of α-diversity indices between study groups. The α-diversity metrics for the microbiomes of the study groups were calculated based on observed OTUs (**A**), Chao1 index (**B**), ACE index (**C**), Shannon index (**D**), and Simpson index (**E**). Differences between groups were determined using the Wilcoxon rank-sum test. OTUs, operational taxonomic units; ACE index, abundance-based coverage estimator.

**Figure 2:**
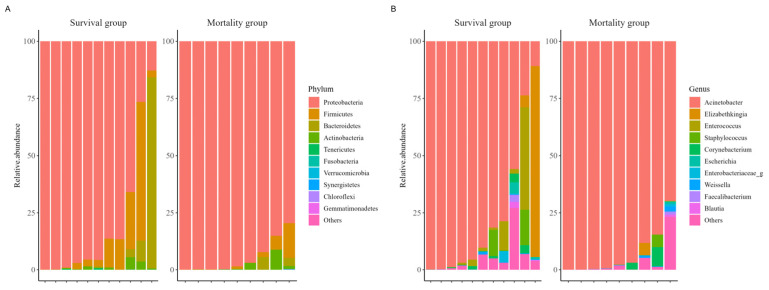
Comparison of composition in relative abundance between study groups among the ten most abundant species. Phylum (**A**), Genus (**B**).

**Figure 3:**
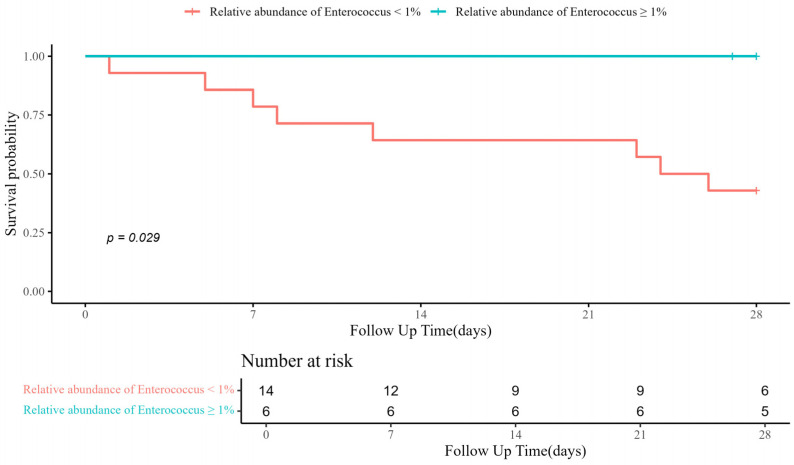
Kaplan–Meier curve for 28-day mortality after diagnosis of CRAB VAP. CRAB VAP, Carbapenem-resistant *Acinetobacter baumannii* ventilator-associated pneumonia.

When comparing α-diversity indices between the survival group and the mortality group, no significant differences were observed in OTUs (43.0 [IQR: 38.5–53.5] vs. 43.0 [IQR: 29.0–79.0]; *p* > 0.99), Chao1 index (63.4 [IQR: 55.5–92.6] vs. 57.2 [IQR: 38.4–114.2]; *p* = 0.656), ACE index (82.0 [IQR: 58.5–112.5] vs. 77.2 [IQR: 56.8–131.2]; *p* > 0.99), Shannon index (0.6 [IQR: 0.2–0.8] vs. 0.1 [IQR: 0.0–0.6]; *p* = 0.287), and Simpson index (0.8 [IQR: 0.7–1.0] vs. 1.0 [IQR: 0.8–1.0]; *p* = 0.182) (Figure 1). The study groups could not be distinguished in the PCoA used to assess β-diversity (Figure S1). Figure 2 presents a comparison of the relative abundance between study groups by each sample. The dominant phylum was *Proteobacteria* (95.5% [IQR: 76.1–98.0] vs. 98.4% [IQR: 92.2–99.8]; *p* = 0.261), and the dominant genus was *Acinetobacter* (90.2% [IQR: 67.3–97.8] vs. 97.7% [IQR: 88.1–99.6]; *p* = 0.201). Firth’s logistic regression analysis was conducted to identify factors associated with ICU mortality or 28-day mortality after ICU discharge (Table 2). In univariate analysis, a relative abundance of the *Enterococcus* genus ≥ 1% and a higher Charlson comorbidity index were statistically significant. Multivariable Firth’s logistic regression further confirmed that a relative abundance of the *Enterococcus* genus ≥ 1% was significantly associated with ICU mortality or 28-day mortality after ICU discharge (odds ratio: 0.06; 95% confidence interval: 0.00–0.771; *p* = 0.029). The Kaplan–Meier survival curve for 28-day mortality following CRAB VAP diagnosis also revealed a significant difference among the study population based on the relative abundance of the *Enterococcus* genus ≥ 1% (*p* = 0.029, log-rank) (Figure 3).

## 4. Discussion

In this study, we analyzed the composition and characteristics of the respiratory microbiome in patients with CRAB VAP. Our study is among the limited research providing respiratory microbiome analysis from CRAB VAP specimens and, uniquely, attempts to present the prognostic value of microbiome findings in this patient group. Most patients exhibited a dominance of the *Acinetobacter* genus; however, in some patients, other genera, such as *Elizabethkingia* or *Enterococcus*, were more abundant than *Acinetobacter*, despite not being identified in conventional cultures. Identifying the causative bacteria is an essential step in VAP treatment. Clinicians typically administer empirical antibiotics first and later adjust treatment based on pathogen identification through culture studies. However, conventional cultures require 48–72 h for results and are often unable to rapidly identify the detected pathogens [3]. In contrast, 16S rRNA gene analysis has been suggested to provide faster results with high accuracy [16,17]. Additionally, 16S rRNA sequencing can sometimes reveal unexpected bacterial dominance in respiratory specimens that tested negative using conventional culture methods [17,18]. Consequently, 16S rRNA gene analysis may aid in the rapid identification of pathogens. Since some patients in our study exhibited discrepancies between conventional culture results and 16S rRNA sequencing, our findings suggest that 16S rRNA sequencing could play a role in treating patients with VAP.

In our study, there was no significant difference in α-diversity indices, including the Shannon index, between the study groups. In contrast, previous studies reported an association between lower microbial diversity and the severity and prognosis of pneumonia [19,20]. However, these studies differ from ours, as they did not focus specifically on VAP or pneumonia caused by a single pathogen. Limited research has been conducted on the respiratory microbiomes of patients with VAP caused by specific pathogens. Qi et al. analyzed the respiratory microbiome of patients with VAP caused by *Pseudomonas aeruginosa* and reported no significant difference in microbial composition and Shannon diversity index between the survivor and non-survivor groups [21]. Yoon et al. reported a lower Shannon index in patients with CRAB pneumonia than in those with non-CRAB pneumonia on mechanical ventilation [22]. Similarly, Xiao et al. also reported lower respiratory microbiome diversity in patients with CRAB VAP than in those on mechanical ventilation with and without CRAB colonization [23]. However, since these studies focused on comparing CRAB VAP with non-CRAB VAP and did not analyze the prognostic factors, our study provides a novel perspective.

The unexpected finding of this study was the protective effect of the relative abundance of the *Enterococcus* genus in the multivariable Firth’s logistic regression analysis. *Enterococcus* is one of the prevalent pathogens of VAP [1], and previous studies have reported its enrichment in the respiratory microbiome of patients with acute respiratory distress syndrome and VAP [18,24]. However, the observed protective effect of *Enterococcus*, a known lower respiratory tract pathogen, on mortality in CRAB VAP is challenging to explain based on existing research. Additionally, Adukauskiene et al. reported a higher mortality rate among the patients with monomicrobial VAP caused by multidrug-resistant *A. baumannii* compared to polymicrobial VAP, and there might be similar underlying mechanisms found in our study [25]. While the authors did not identify the exact mechanism behind the worse prognosis of monomicrobial VAP, they suggested that polymicrobial infection might be less virulent due to pathogen competition. Our findings may also be explained by pathogen competition during infection. Furthermore, one study reported that *Enterococcus faecalis* can inhibit the growth of *Pseudomonas aeruginosa* [26]. The bacteriocins (enterocins) produced by *Enterococcus* species may also possess antimicrobial activity and could have contributed to this result [27,28].

This study has some limitations. First, as a single-center study with a small study population, our findings may not be generalizable to the broader population of patients with CRAB VAP. Nevertheless, our study adds valuable insights to the literature, given the scarcity of microbiome research in patients with CRAB VAP. Second, including patients with non-CRAB VAP as a comparator group in our analysis could have provided a more comprehensive understanding of respiratory microbiome dynamics. Third, we were unable to assess temporal changes in the respiratory microbiome during the treatment, as our study focused solely on respiratory specimens collected at the time of CRAB VAP diagnosis. Fourth, all cases of CRAB VAP in our study occurred during antibiotic therapy, and previously administered antibiotics may have influenced the microbiome analysis.

## 5. Conclusions

In conclusion, our study characterized the respiratory microbiome in patients with CRAB VAP and suggested that microbiome analysis, particularly the relative abundance of genus-level organisms, may have prognostic value in this condition. Therefore, further studies with larger sample sizes are warranted to validate these findings.

## Figures and Tables

**Table 1:** Baseline characteristics and treatment outcomes of the study population.

	Study Population (N = 20)	Survival Group (N = 11)	Mortality Group (N = 9)	*p*-Value
Age, years	69.0 (61.5–79.0)	62.0 (56.5–75.5)	72.0 (66.0–81.0)	0.254
Age ≥ 65 years	13 (65.0%)	5 (45.5%)	8 (88.9%)	0.120
Sex, male	13 (65.0%)	9 (81.8%)	4 (44.4%)	0.203
BMI, kg/m^2^	22.9 (20.4–26.1)	21.9 (21.0–25.1)	24.4 (19.3–26.1)	0.820
Comorbidities				
Hypertension	5 (25.0%)	4 (36.4%)	1 (11.1%)	0.436
Cerebrovascular accident	3 (15.0%)	2 (18.2%)	1 (11.1%)	>0.99
Diabetes mellitus	8 (40.0%)	5 (45.5%)	3 (33.3%)	0.927
Chronic kidney disease	4 (20.0%)	2 (18.2%)	2 (22.2%)	>0.99
Chronic liver disease	1 (5.0%)	0	1 (11.1%)	>0.918
Asthma	1 (5.0%)	1 (9.1%)	0	>0.99
Bronchiectasis	2 (10.0%)	1 (9.1%)	1 (11.1%)	>0.99
COPD	5 (25.0%)	2 (18.2%)	3 (33.3%)	0.795
Interstitial lung disease	3 (15.0%)	1 (9.1%)	2 (22.2%)	0.850
Cancer	7 (35.0%)	2 (18.2%)	5 (55.6%)	0.203
Connective tissue disease	2 (10.0%)	0	2 (22.2%)	0.369
Charlson comorbidity index	5.0 (3.0–7.0)	3.0 (2.5–5.0)	7.0 (6.0–9.0)	0.024
Mechanical ventilation days before the diagnosis of CRAB VAP	14.0 (9.0–31.5)	14.0 (9.5–21.5)	14.0 (10.0–36.0)	0.675
Vasopressor during ICU stay	20 (100.0%)	11 (100.0%)	9 (100.0%)	
Prone position during ICU stay	1 (5.0%)	1 (9.1%)	0	>0.99
Continuous renal replacement therapy	4 (20.0%)	1 (9.1%)	3 (33.3%)	0.432
ECMO	2 (10.0%)	2 (18.2%)	0	0.549
SOFA score	6.0 (4.5–7.0)	6.0 (3.5–7.0)	6.0 (5.0–8.0)	0.490
Laboratory test				
White blood cell, /μL	9800 (8200–15000)	9.3 (8.2–12.0)	14.2 (9.1–18.8)	0.201
Lymphocyte count, /μL	900 (500–1200)	1000.0 (915.0–1525.0)	600.0 (260.0–1010.0)	0.046
Hemoglobin, g/dL	8.6 (7.7–9.2)	8.6 (7.7–8.9)	8.8 (8.3–10.0)	0.543
Platelet count, 10^3^/μL	255.0 (165.0–369.5)	253.0 (198.0–412.5)	271.(92.0–295.0)	0.305
Blood urea nitrogen, mg/dL	26.1 (11.9–39.5)	20.9 (11.9–31.6)	36.0 (22.9–46.1)	0.224
Creatinine, mg/dL	0.6 (0.4–0.9)	0.6 (0.4–0.7)	0.7 (0.4–0.9)	0.820
Arterial lactate, mmol/L	1.6 (0.9–1.9)	1.5 (0.9–1.7)	1.9 (1.1–2.9)	0.177
C-reactive protein, mg/L	64.5 (31.6–110.0)	59.9 (33.4–79.7)	82.9 (32.8–123.6)	0.370
Antibiotics on sampling date				
Cephalosporin	1 (5.0%)	1 (9.1%)	0	>0.99
Penicillin	7 (35.0%)	4 (36.4%)	3 (33.3%)	>0.99
Carbapenem	14 (70.0%)	7 (63.6%)	7 (77.8%)	0.844
Quinolone	9 (45.0%)	5 (45.5%)	4 (44.4%)	>0.99
Aminoglycoside	3 (15.0%)	1 (9.1%)	2 (22.2%)	0.850
Colistin	6 (30.0%)	2 (18.2%)	4 (44.4%)	0.433
Glycopeptide	12 (60.0%)	9 (81.8%)	3 (33.3%)	0.081
Outcomes				
In-ICU mortality or mortality within 28 days of ICU discharge	9 (45.0%)	0	9 (100%)	<0.001
In-ICU mortality	5 (25.0%)	0	5 (55.6%)	0.02
28-day mortality	8 (40.0%)	0	8 (88.9%)	<0.001
60-day mortality	10 (50.0%)	1 (9.1%)	9 (100.0%)	<0.001
In-hospital mortality	11 (55.0%)	2 (18.2%)	9 (100.0%)	0.001

BMI, body mass index; COPD, chronic obstructive pulmonary disease; CRAB VAP, carbapenem-resistant *Acinetobacter baumannii* ventilator-associated pneumonia; ICU, intensive care unit; ECMO, extracorporeal membrane oxygenation; SOFA, Sequential Organ Failure Assessment.

**Table 2:** Risk factors for ICU mortality or 28-day mortality after ICU discharge in patients with CRAB VAP.

	Univariate Analysis				Multivariable Analysis			
	OR	2.5%	97.5%	*p*-Value	OR	2.5%	97.5%	*p*-Value
Relative abundance of *Enterococcus* genus ≥ 1%	0.04	0.00	0.50	0.008	0.06	<0.001	0.77	0.029
Charlson comorbidity index	1.52	1.07	2.46	0.018	1.43	0.97	2.41	0.070
Age ≥ 65 years	6.70	1.00	78.5	0.05				
Lymphocyte count < 1000/μL	2.19	0.40	13.47	0.367				
Male sex	0.22	0.03	1.29	0.094				
*Proteobacteria* to *Firmicutes* ratio	1.00	1.00	1.00	0.181				
*Proteobacteria* to *Bacteroidetes* ratio	1.00	1.00	1.00	0.200				
*Firmicutes* to *Bacteroidetes* ratio	1.00	0.99	1.00	0.248				
SOFA score	1.12	0.85	1.55	0.421				

ICU, intensive care unit; CRAB VAP, Carbapenem-resistant *Acinetobacter baumannii* ventilator-associated pneumonia; SOFA, Sequential Organ Failure.

## Data Availability

The data that support the findings of this study are not openly available due to reasons of sensitivity and are available from the corresponding author upon reasonable request. Data are located in controlled access data storage at Yonsei University College of Medicine.

## Supplementary Materials

The following supporting information can be downloaded at: https://www.mdpi.com/article/10.3390/pathogens14111141/s1, Figure S1: Principal coordinate analysis to evaluate the β-diversity of each group of bacteria.

## Abbreviations

The following abbreviations are used in this manuscript:

ACE	abundance-based coverage estimator
CRAB	Carbapenem-resistant *A. baumannii*
ICU	intensive care unit
IQR	interquartile range
OTU	operational taxonomic unit
PCoA	principal coordinate analysis
PCR	polymerase chain reaction
SOFA	Sequential Organ Failure Assessment
VAP	Ventilator-associated pneumonia
