# Supplementary material for: Respiratory Microbiome of Carbapenem-Resistant Acinetobacter baumannii Ventilator-Associated Pneumonia: A Pilot Study from the Republic of Korea
Source: Pathogens. 2025 Nov 11;14(11):1141. doi: 10.3390/pathogens14111141 (PMC12655194; doi:10.3390/pathogens14111141)
Supplement: Supplementary file 1 [file pathogens-14-01141-s001.zip › pathogens-3904205-supplementary.pdf]

Respiratory microbiome of carbapenem-resistant *Acinetobacter baumannii* ventilator associated pneumonia: A Pilot Study from the Republic of Korea

Supplementary material

Figure S1 Principal coordinate analysis to evaluate the β-diversity of each group of bacteria.

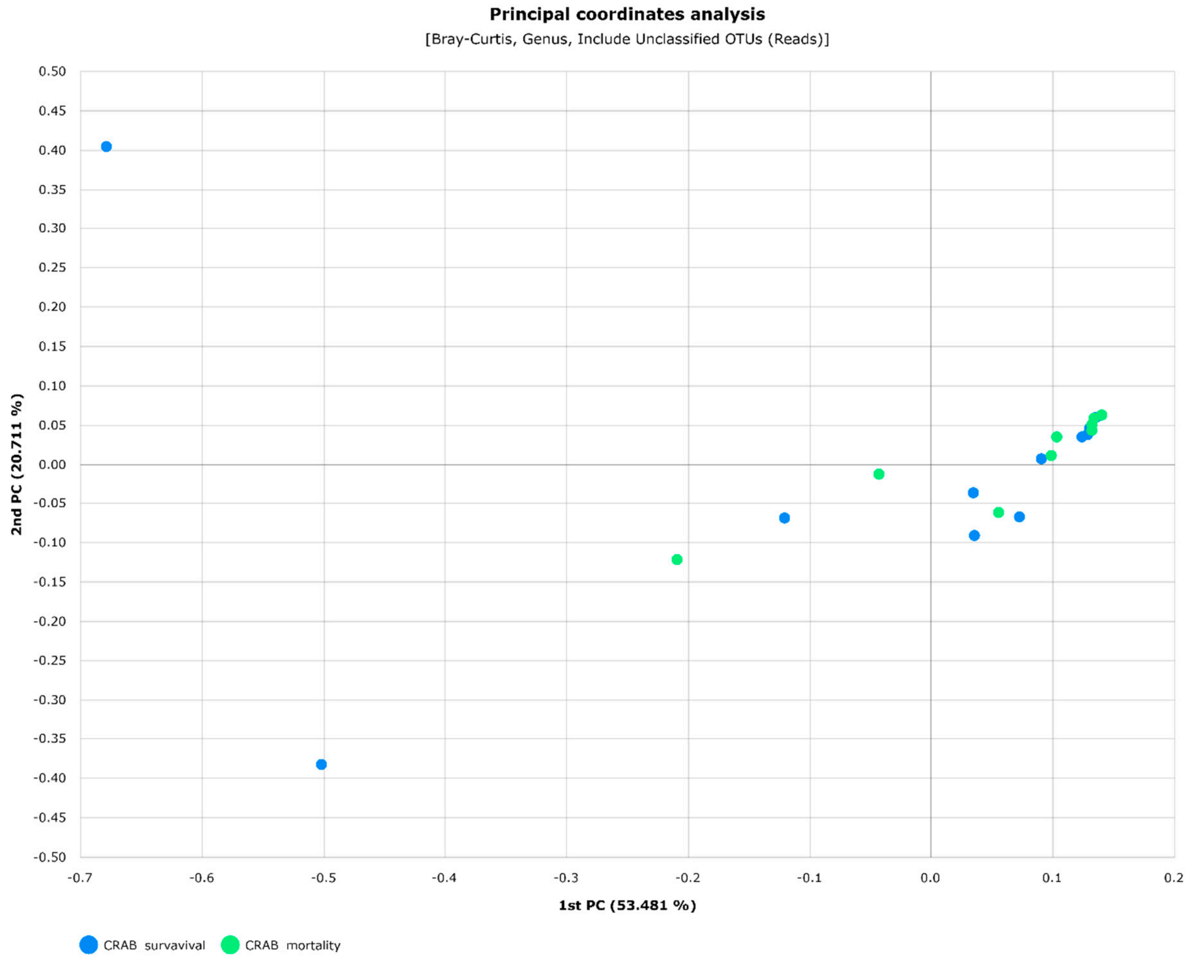

Beta diversity of microbiome composition from endotracheal aspirate (ETA) samples of the study population. The principal coordinate plots are based on the Bray-Curtis distance according to the mortality group (blue) and survivor group (green). Points represent a single ETA from a single individual in each group.
